# Supplementary material for: Resting-state fMRI signals contain spectral signatures of local hemodynamic response timing
Source: eLife. 2023 Aug 11;12:e86453. doi: 10.7554/eLife.86453 (PMC10506795; doi:10.7554/eLife.86453)
Supplement: Supplementary file 1. [file elife-86453-supp1.docx]

*Table S1:* *p-values for all subjects and all resting-state spectral features between fast, slow, and LGN voxels, within individual subjects.* Significant differences based on Wilcoxon rank-sum test (p<0.05) are bolded.

|  | *Slope < 0.2 Hz* | | | *Aperiodic Exponent* | | | *ALFF* | | | *fALFF* | | |
| --- | --- | --- | --- | --- | --- | --- | --- | --- | --- | --- | --- | --- |
|  | Fast-Slow | Fast-LGN | Slow-LGN | Fast-Slow | Fast-LGN | Slow-LGN | Fast-Slow | Fast-LGN | Slow-LGN | Fast-Slow | Fast-LGN | Slow-LGN |
| S1 | **1.13E-14** | **6.51E-09** | **7.15E-17** | **1.13E-12** | **3.26E-09** | **1.71E-16** | **6.98E-14** | 0.7237 | **7.52E-09** | **1.64E-14** | **7.88E-10** | **7.38E-17** |
| S2 | **1.29E-21** | **3.93E-10** | **6.16E-14** | **6.21E-22** | **6.12E-11** | **9.59E-14** | **2.33E-10** | **0.0021** | 0.4402 | **2.59E-20** | **3.26E-05** | **1.14E-10** |
| S3 | **5.06E-16** | **3.08E-06** | **1.13E-10** | **7.46E-17** | **4.79E-06** | **5.69E-11** | **4.89E-05** | 0.8869 | **0.0159** | **3.34E-15** | **7.78E-07** | **6.76E-11** |
| S4 | **3.51E-03** | **4.42E-06** | **2.06E-08** | **3.95E-03** | **3.82E-07** | **2.59E-09** | **3.20E-03** | 0.2091 | **0.0079** | 0.0643 | **2.09E-05** | **2.05E-07** |
| S5 | **6.67E-08** | **2.88E-09** | **6.29E-10** | **3.91E-12** | **1.17E-09** | **3.45E-10** | **1.15E-05** | **2.98E-05** | **0.0446** | **2.02E-06** | **6.62E-09** | **8.86E-10** |
| S6 | **3.61E-25** | **1.01E-10** | **1.85E-12** | **4.20E-22** | **1.12E-10** | **1.34E-12** | **3.59E-20** | 0.9969 | **6.20E-06** | **3.72E-30** | **9.55E-07** | **1.27E-11** |
| S7 | **9.71E-04** | **2.51E-04** | **7.79E-05** | 0.1432 | **2.51E-04** | **9.19E-05** | 0.5493 | **0.0498** | 0.0867 | **0.0322** | **3.76E-04** | **1.75E-04** |
| S8 | **6.89E-14** | **1.39E-09** | **4.15E-14** | **1.20E-20** | **1.74E-09** | **1.41E-14** | **3.14E-03** | 0.4995 | 0.2247 | **4.56E-11** | **1.48E-09** | **9.87E-14** |
| S9 | **1.72E-24** | **9.55E-15** | **7.08E-21** | **6.84E-18** | **1.64E-14** | **6.48E-21** | **0.5928** | 0.6579 | 0.2915 | **1.27E-24** | **5.91E-11** | **8.89E-20** |
| S10 | **4.47E-10** | **1.75E-13** | **9.63E-19** | **9.19E-04** | **9.38E-18** | **9.81E-22** | 0.1464 | 0.3322 | 0.8048 | **9.32E-10** | **5.00E-13** | **5.58E-18** |
| S11 | **6.65E-04** | **2.19E-11** | **6.32E-12** | **4.96E-04** | **1.39E-11** | **4.80E-12** | **6.78E-04** | 0.0585 | 0.1637 | 0.0533 | **1.05E-09** | **4.39E-10** |
| S12 | **4.08E-13** | **1.41E-10** | **3.68E-13** | **1.77E-15** | **4.72E-09** | **1.01E-12** | **1.83E-09** | **5.88E-08** | **1.15E-10** | **1.83E-07** | **1.03E-09** | **2.38E-12** |
| S13 | **8.83E-10** | **7.77E-08** | **1.79E-11** | **5.32E-09** | **4.31E-04** | **8.21E-09** | 0.3715 | **4.92E-10** | **5.66E-07** | **8.67E-08** | **9.20E-10** | **2.47E-12** |
| S14 | **5.76E-04** | **4.19E-06** | **1.54E-08** | **2.70E-03** | **5.63E-06** | **8.22E-09** | 0.3232 | 0.1669 | 0.3733 | **0.0233** | **5.27E-06** | **9.18E-08** |
| S15 | **1.92E-12** | **1.06E-07** | **9.38E-11** | **6.07E-14** | **1.44E-08** | **2.79E-11** | **1.81E-07** | **8.08E-04** | 0.8278 | **8.47E-13** | **8.46E-05** | **2.32E-09** |
