# Supplementary material for: Resting-state fMRI signals contain spectral signatures of local hemodynamic response timing
Source: eLife. 2023 Aug 11;12:e86453. doi: 10.7554/eLife.86453 (PMC10506795; doi:10.7554/eLife.86453)
Supplement: Supplementary file 2. [file elife-86453-supp2.docx]

*Table S2: Average Classification Accuracies per subject.* Results are average of over 1000 bootstraps with 95% confidence intervals for each subject on each model trained. For each subject the model with the highest accuracy is bolded. Chance is 33%.

|  | Subsampled Spectra | Spectral Features | Breath Hold Latencies |
| --- | --- | --- | --- |
| S1 | **71.43 % (0.31)** | 65.21 % (0.37) | 58.46 % (0.36) |
| S2 | 68.91 % (0.31) | **71.63 % (0.31)** | 50.08 % (0.34) |
| S3 | **70.46 % (0.44)** | 69.25 % (0.46) | 49.06 % (0.50) |
| S4 | **58.63 % (0.49)** | 53.88 % (0.47) | 38.39 % (0.45) |
| S5 | **62.48 % (0.30)** | 62.19 % (0.31) | 52.94 % (0.35) |
| S6 | **80.56 % (0.25)** | 77.37 % (0.29) | 60.19 % (0.34) |
| S7 | **72.69 % (0.48)** | 59.31 % (0.54) | 64.01 % (0.53) |
| S8 | **73.18 % (0.35)** | 72.12 % (0.37) | 42.57 % (0.38) |
| S9 | 72.87 % (0.29) | **74.38 % (0.29)** | 53.93 % (0.34) |
| S10 | **73.54 % (0.24)** | 61.90 % (0.26) | 49.16 % (0.26) |
| S11 | 50.09 % (0.44) | **59.49 % (0.43)** | 46.74 % (0.45) |
| S12 | 63.63 % (0.33) | **64.98 % (0.34)** | 51.65 % (0.37) |
| S13 | **67.50 % (0.32)** | 67.14 % (0.35) | 46.58 % (0.35) |
| S14 | **57.92 % (0.37)** | 57.34 % (0.38) | 50.83 % (0.36) |
| S15 | **67.99 % (0.35)** | 61.94 % (0.36) | 63.08 % (0.39) |
| COMBINED | **66.68 % (0.08)** | 65.70 % (0.08) | 52.02 % (0.11) |
