# Supplementary material for: Resting-state fMRI signals contain spectral signatures of local hemodynamic response timing
Source: eLife. 2023 Aug 11;12:e86453. doi: 10.7554/eLife.86453 (PMC10506795; doi:10.7554/eLife.86453)
Supplement: Supplementary file 3. [file elife-86453-supp3.docx]

*Table S3. Subject-wise results from fit of linear model relating each spectral feature with phase. p-values are from a linear hypothesis test on the model coefficients.*

|  | *Slope < 0.2 Hz* | | | *Aperiodic Exponent* | | | *ALFF* | | | *fALFF* | | |
| --- | --- | --- | --- | --- | --- | --- | --- | --- | --- | --- | --- | --- |
|  | **x1** | **R^2^** | **p-value** | **x1** | **R^2^** | **p-value** | **x1** | **R^2^** | **p-value** | **x1** | **R^2^** | **p-value** |
| S1 | 0.3865 | 0.1058 | 1.51E-16 | 0.3391 | 0.0781 | 1.93E-12 | 0.3998 | 0.1063 | 1.28E-16 | 0.3868 | 0.1080 | 7.14E-17 |
| S2 | 0.3657 | 0.1204 | 1.46E-20 | 0.4044 | 0.1251 | 2.40E-21 | 0.2475 | 0.0500 | 4.24E-09 | 0.3239 | 0.1017 | 1.93E-17 |
| S3 | 0.3935 | 0.2451 | 4.94E-42 | 0.3940 | 0.2457 | 1.73E-40 | 0.2096 | 0.0621 | 2.10E-10 | 0.3691 | 0.2533 | 6.81E-42 |
| S4 | 0.2592 | 0.1153 | 5.87E-17 | 0.2692 | 0.1208 | 9.75E-18 | 0.1670 | 0.0351 | 6.14E-06 | 0.2188 | 0.0922 | 1.04E-13 |
| S5 | 0.1836 | 0.0413 | 1.93E-09 | 0.2591 | 0.0718 | 1.49E-15 | 0.1394 | 0.0227 | 9.65E-06 | 0.1727 | 0.0364 | 1.81E-08 |
| S6 | 0.3990 | 0.2049 | 6.30E-44 | 0.3721 | 0.1695 | 6.70E-36 | 0.3041 | 0.1270 | 1.07E-26 | 0.4249 | 0.2455 | 1.47E-53 |
| S7 | 0.2918 | 0.1363 | 1.09E-27 | 0.2558 | 0.0911 | 1.34E-18 | 0.1839 | 0.0710 | 1.09E-14 | 0.3188 | 0.1625 | 3.73E-33 |
| S8 | 0.2714 | 0.1870 | 8.47E-37 | 0.2969 | 0.2009 | 1.00E-39 | 0.1327 | 0.0593 | 5.78E-12 | 0.2725 | 0.1868 | 9.43E-37 |
| S9 | 0.3289 | 0.2446 | 5.36E-43 | 0.2775 | 0.1641 | 1.31E-27 | -0.0130 | 3.28E-4 | 6.38E-01 | 0.3258 | 0.2519 | 2.01E-44 |
| S10 | 0.2136 | 0.0618 | 2.87E-16 | 0.1492 | 0.0277 | 5.86E-08 | -0.0320 | 0.0013 | 2.41E-01 | 0.2145 | 0.0648 | 5.10E-17 |
| S11 | 0.2033 | 0.0509 | 6.62E-13 | 0.1769 | 0.0328 | 9.44E-09 | 0.0555 | 0.0032 | 7.59E-02 | 0.1810 | 0.0424 | 5.78E-11 |
| S12 | 0.2287 | 0.0797 | 2.62E-18 | 0.2434 | 0.0855 | 1.42E-19 | 0.1593 | 0.0351 | 1.06E-08 | 0.1814 | 0.0527 | 1.85E-12 |
| S13 | 0.2697 | 0.0905 | 6.89E-20 | 0.2490 | 0.0735 | 2.60E-16 | 0.1060 | 0.0127 | 8.16E-04 | 0.2467 | 0.0793 | 1.60E-17 |
| S14 | 0.1062 | 0.0153 | 3.11E-04 | 0.0883 | 0.0103 | 3.08E-03 | 0.0462 | 0.0030 | 1.13E-01 | 0.0592 | 0.0049 | 4.19E-02 |
| S15 | 0.2537 | 0.0818 | 6.84E-17 | 0.2710 | 0.0892 | 2.40E-18 | 0.1104 | 0.0159 | 2.99E-04 | 0.2268 | 0.0668 | 5.75E-14 |
