# Supplementary material for: Resting-state fMRI signals contain spectral signatures of local hemodynamic response timing
Source: eLife. 2023 Aug 11;12:e86453. doi: 10.7554/eLife.86453 (PMC10506795; doi:10.7554/eLife.86453)
Supplement: Supplementary file 4. [file elife-86453-supp4.docx]

*Table S4: Average regression coefficient of determination (*$R^{2})$ *and Root Mean Squared Error (RMSE)*. Results are averaged over 1000 bootstraps with 95% confidence intervals for each subject on each model trained. All RMSE values are smaller than the RMSE from a model trained on shuffled labels.

|  | *R^2^* | | *RMSE* | |
| --- | --- | --- | --- | --- |
|  | **Subsampled Spectra** | **Spectral Features** | **Subsampled Spectra** | **Spectral Features** |
| S1 | 0.203 (0.006) | 0.057 (0.005) | 0.724 (0.003) | 0.780 (0.003) |
| S2 | -0.012 (0.006) | 0.052 (0.005) | 0.822 (0.004) | 0.793 (0.004) |
| S3 | 0.422 (0.006) | 0.237 (0.005) | 0.961 (0.005) | 0.971 (0.004) |
| S4 | 0.173 (0.008) | 0.067 (0.005) | 1.217 (0.006) | 1.095 (0.004) |
| S5 | -0.041 (0.005) | -0.036 (0.004) | 0.914 (0.003) | 0.880 (0.003) |
| S6 | 0.278 (0.005) | 0.242 (0.004) | 0.896 (0.004) | 0.924 (0.004) |
| S7 | 0.326 (0.008) | 0.167 (0.005) | 0.913 (0.005) | 1.015 (0.004) |
| S8 | 0.266 (0.006) | 0.173 (0.004) | 1.112 (0.004) | 1.151 (0.004) |
| S9 | 0.389 (0.004) | 0.342 (0.005) | 1.065 (0.004) | 1.106 (0.004) |
| S10 | 0.253 (0.004) | 0.060 (0.004) | 0.909 (0.003) | 1.022 (0.003) |
| S11 | -0.078 (0.008) | 0.002 (0.003) | 0.787 (0.004) | 0.914 (0.003) |
| S12 | 0.120 (0.006) | 0.049 (0.004) | 0.999 (0.004) | 1.033 (0.003) |
| S13 | 0.147 (0.005) | 0.104 (0.004) | 0.903 (0.003) | 0.928 (0.003) |
| S14 | 0.149 (0.007) | -0.023 (0.003) | 0.790 (0.004) | 0.947 (0.003) |
| S15 | 0.197 (0.005) | 0.044 (0.004) | 0.803 (0.003) | 0.927 (0.003) |
| COMBINED | 0.171 (0.001) | 0.123 (0.001) | 0.949 (0.002) | 0.976 (0.001) |
