# Supplementary material for: Resting-state fMRI signals contain spectral signatures of local hemodynamic response timing
Source: eLife. 2023 Aug 11;12:e86453. doi: 10.7554/eLife.86453 (PMC10506795; doi:10.7554/eLife.86453)
Supplement: Supplementary file 5. [file elife-86453-supp5.docx]

*Table S5****.*** *Simulated HRF parameters.*

|  | TTP (s) | FWHM (s) | Peak PSC  (BOLD % Signal Change) |
| --- | --- | --- | --- |
| HRF #1 | 6.0 | 5 | 6.0 |
| HRF #2 | 4.5 | 3.5 | 5.0 |
| HRF #3 | 3.5 | 3.0 | 2.5 |
| HRF #4 | 3.0 | 3.0 | 2.0 |
| HRF #5 | 2.5 | 2.0 | 1.5 |
| HRF #6 | 6.5 | 5.5 | 1.0 |
